# Real-World Performance of the 2026 AHA/ACC Pulmonary Embolism Framework in a Multi-System CTPA Cohort

**DOI:** 10.64898/2026.08.06.26359865

**Published:** 2026-08-10

**Authors:** Mahmoud Alwakeel, Suraj Zaveri, Emory Buck, Sudarshan Rajagopal, Divya Verma, Daniel Loriaux, Ricardo Henao, Victor F. Tapson, Thomas L. Ortel, William Schuyler Jones, Jon Martin, Krista L. Haines, Nikki L. B. Freeman, An-Kwok Ian Wong

## Abstract

**Background:**

The 2026 American Heart Association/American College of Cardiology (AHA/ACC) guidelines replaced the 2019 European Society of Cardiology (ESC) four-tier pulmonary embolism (PE) risk scheme with five clinical categories (A–E) and subcategories. These categories were set by expert consensus and have not been validated against outcomes. How patients are reclassified relative to ESC, or how the two systems compare prognostically, is unknown.

**Methods:**

We utilized three cohorts of patients with confirmed PE using structured electronic health record data, laboratory biomarkers, and large-language-model abstraction of radiology reports: Duke University Health System (n=12,992, drawn from 95,760 consecutive inpatient CT pulmonary angiography studies, 2014–2025, with no referral or registry enrollment step between imaging and cohort entry), INSPECT (Stanford; n=3,870), and MIMIC-IV (Beth Israel Deaconess; n=361). Patients were assigned AHA/ACC categories B through E, subcategorized where data allowed, and mapped to 2019 ESC risk strata. The primary outcome was 30-day mortality; discrimination was assessed with Harrell C-index.

**Results:**

Among 17,223 patients with confirmed PE, pooled 30-day mortality rose monotonically across categories: 1.5% (B), 8.9% (C), 15.5% (D), and 31.9% (E), with the ordering preserved in all three cohorts despite differing baseline mortality. Subcategory-level discrimination was reliable only at the high-acuity extreme (D2–E2); across subcategories C1 through D1, mortality did not order monotonically (9.2%, 10.8%, 8.1%, 10.9%), and adding subcategories to category C did not improve discrimination at Duke (C-index 0.699 vs 0.699). Category C patients lacking both echocardiography and biomarker testing (12.7% of category C) had mortality (10.4%) equal to or exceeding classified peers. Relative to ESC, the frameworks were concordant at the extremes, but 5.7%of ESC intermediate-risk patients were reclassified to category D, with modestly higher but non-significant 30-day mortality than those remaining in category C (10.8% versus 8.9%).

**Conclusions:**

Across a three-health-system cohort, the 2026 AHA/ACC framework produced a reproducible mortality gradient at the category level, with added subcategory granularity refining risk chiefly at the highest-acuity tiers. Discrimination across the broad intermediate band was limited, and reclassification from ESC fell almost entirely within this range.

## Full Text Availability

The license terms selected by the author(s) for this preprint version do not permit archiving in PMC. The full text is available from the preprint server.

